# SKN-1 regulates stress resistance downstream of amino catabolism pathways

**DOI:** 10.1016/j.isci.2022.104571

**Published:** 2022-06-09

**Authors:** Phillip A. Frankino, Talha F. Siddiqi, Theodore Bolas, Raz Bar-Ziv, Holly K. Gildea, Hanlin Zhang, Ryo Higuchi-Sanabria, Andrew Dillin

**Affiliations:** 1 Howard Hughes Medical Institute University of California, Berkeley, CA 94720, USA; 2 California Institute for Regenerative Medicine, Berkeley, CA 94720, USA; 3 Helen Wills Neuroscience Institute, Berkeley, CA 94720, USA; 4 Department of Molecular and Cell Biology, University of California, Berkeley, Berkeley, CA, USA; 5 Leonard Davis School of Gerontology, University of Southern California, Los Angeles, CA 90089, USA

**Keywords:** Biological sciences, Molecular biology, Transcriptomics

## Abstract

The deleterious potential to generate oxidative stress is a fundamental challenge to metabolism. The oxidative stress response transcription factor, SKN-1/NRF2, can sense and respond to changes in metabolic state, although the mechanism and consequences of this remain unknown. Here, we performed a genetic screen in *C. elegans* targeting amino acid catabolism and identified multiple metabolic pathways as regulators of SKN-1 activity. We found that knockdown of the conserved amidohydrolase *T12A2.1/amdh-1* activates a unique subset of SKN-1 regulated genes. Interestingly, this transcriptional program is independent of canonical P38-MAPK signaling components but requires ELT-3, NHR-49 and MDT-15. This activation of SKN-1 is dependent on upstream histidine catabolism genes HALY-1 and Y51H4A.7/UROC-1 and may occur through accumulation of a catabolite, 4-imidazolone-5-propanoate. Activating SKN-1 results in increased oxidative stress resistance but decreased survival to heat stress. Together, our data suggest that SKN-1 acts downstream of key catabolic pathways to influence physiology and stress resistance.

## Highlights

• Genetic perturbation of amino acid catabolism pathways leads to SKN-1 activity
• Nutrient sensing and signaling pathways are partially required
• Buildup of a histidine catabolite (4-imidazolone-5-propionate) likely activates SKN-1
• SKN-1 activation results in differential stress resilience

## Graphical abstract

**Figure:**
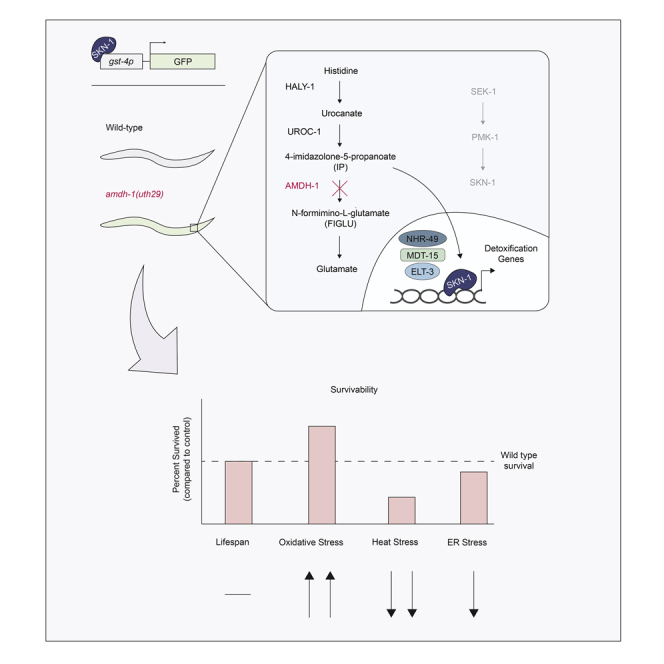

## Introduction

Metabolism is central to normal cell function and is dysregulated in human diseases such as metabolic syndrome, diabetes, and cancer (DeBerardinis and Thompson, 2012). In the most basic sense, metabolism is the sum of all biochemical reactions in the cell, including reactions that create or break down complex molecules (anabolism and catabolism, respectively). The catabolism of amino acids leads to the accumulation of breakdown products, or catabolites, that are essential for creating cellular energy through the tricarboxylic acid (TCA) cycle and cellular respiration. Outside of their role in creating cellular energy, many of these catabolites have been identified as signaling molecules that affect both normal cell function and disease (Martínez-Reyes and Chandel, 2020). For example, tryptophan catabolites are known immunomodulators, and elevated expression of tryptophan catabolism enzymes is associated with cancer progression and poor prognosis (McGaha et al., 2012). Additionally, the histidine catabolite, imidazolone propionate, is elevated in type 2 diabetic patients and has been shown to impair insulin signaling (Chong et al., 2018; Molinaro et al., 2020). Despite their importance, our understanding of the identity, mechanism and physiological consequences of catabolite signaling is incomplete.

A key challenge for metabolism is the resolution of deleterious byproducts that can damage cellular components. For example, the electron transport chain of the mitochondria is the main site of ATP generation but also produces harmful reactive oxygen species (ROS), a form of oxidative stress that damages DNA, lipid membranes and proteins (Nolfi-Donegan et al., 2020). Cells respond to oxidative stress and damage through multiple complex signaling cascades that upregulate genes to help maintain homeostasis, depending on the type and severity of the stress. Central to these pathways, referred to as the oxidative stress response (OxSR) for simplicity, is the transcription factor SKN-1/NRF2, which binds to antioxidant response elements in the promoters of genes, including detoxification and antioxidant synthesis enzymes, that are important for survival under a wide range of oxidative stressors (Blackwell et al., 2015).

In response to oxidative stress in *Caenorhabditis elegans*, SKN-1 becomes phosphorylated and activated by the conserved map kinase (MAPK) pathway through a signaling cascade that involves the MAPKK and P38/MAPK homologs (SEK-1 and PMK-1, respectively) (Inoue et al., 2005). Once this signaling cascade is initiated, nuclear factors such as ELT-3, NHR-49, and MDT-15 are required for upregulation of stress response targets (Goh et al., 2014, 2018; Hu et al., 2017; Wu et al., 2016). Together, the OxSR alleviates oxidative stress and restores homeostasis to promote cell and organismal survival.

SKN-1 is well known for its role in the OxSR but emerging literature has implicated it as a transcription factor that can respond to changes in metabolism. For example, exogenous supplementation of amino acids activates SKN-1-mediated transcription (Edwards et al., 2015). SKN-1 can also respond to changes in proline catabolism to mobilize lipids during starvation (Pang et al., 2014). Furthermore, genetic perturbation of multiple amino acid catabolic pathways activates SKN-1-mediated transcription (Fisher et al., 2008; Ravichandran et al., 2018; Tang and Pang, 2016). Intriguingly, many of these instances of SKN-1 activation may involve diverse catabolites, suggesting this as a mechanism that works to integrate the state of multiple metabolic pathways and to respond to metabolic imbalance through a single effector. To date, no study has systematically probed amino acid catabolism pathways to better understand their role in SKN-1 activation or the resulting physiological responses.

Here, we identify multiple pathways of amino acid catabolism that, when perturbed, activate a distinct transcriptional response driven by SKN-1. Using a mutant of histidine catabolism as a model, we show that this response is independent of canonical MAPK signaling pathways and may partially depend on GCN2 and mTOR homologs *gcn-2* and *let-363*. We also demonstrate the necessity of nuclear factors previously implicated in the OxSR for SKN-1 activation. Interestingly, this response is dependent on the upstream enzymes of the histidine catabolism pathway, suggesting endogenous catabolites may directly or indirectly activate SKN-1. Activation of SKN-1 via mutation of the histidine catabolism pathway results in increased oxidative stress resistance at the cost of decreased resistance to heat stress, indicating that SKN-1 can differentially influence stress response survival in response to metabolic disruption. Together, our data implicate SKN-1 as a transcriptional effector downstream of amino acid catabolism pathways, which likely becomes activated through accumulation of catabolite intermediates, to control susceptibility to stress.

## Results

### Genetic perturbation of amino acid catabolism pathways activates SKN-1

**Figure 1:**
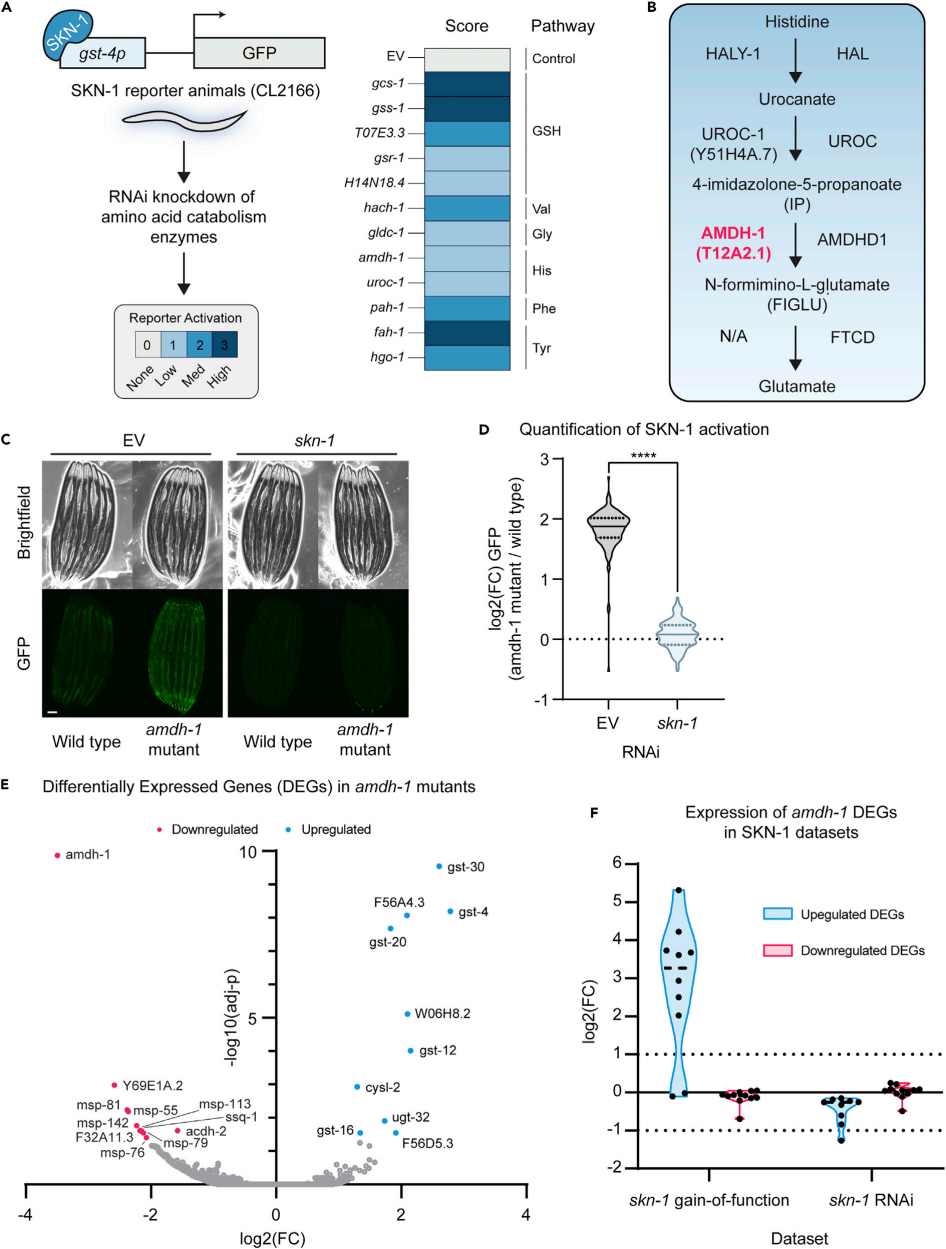
Perturbation of histidine catabolism activates an SKN-1 mediated detoxification response (A) Experimental scheme of RNAi screen to uncover amino acid catabolism enzymes that affect SKN-1 activation (left), and scores of tested genes (right). RNAi knockdown of genes that suppressed the reporter were scored but not included (Table 1). (B) Enzymes and intermediates involved in the histidine catabolism pathway, in *C. elegans* (left) and humans (right) (“N/A” represents no identified *C. elegans* enzyme for this step). (C) Fluorescent images of SKN-1 transcriptional reporter, *gst-4p::GFP,* in wildtype or mutant backgrounds on RNAi Scale bar, 100 μm. (D) Quantification of SKN-1 activation (*amdh-1* mutant normalized to median of wild type) from (C), Data are representative of n = 3 biological replicates, n > 121 animals per replicate, ∗∗∗∗ = p < 0.0001 using a Mann-Whitney two-tailed test. (E) Volcano plot of genes in *amdh-1(uth29)* compared to N2 wildtype control. Differentially expressed genes (DEGs) shown in red (downregulated) and blue (upregulated), adjusted-p < 0.05 (F) Meta analysis of DEGs from *amdh-1(uth29)* mutants in gain of function *skn-1(lax188)* and *skn-1* RNAi datasets (Nhan et al., 2019; Steinbaugh et al., 2015).

To uncover the genetic mechanisms by which amino acid catabolism pathways signal to SKN-1, we performed an RNAi screen to identify the amino acid catabolism pathways that, when disrupted, activate an SKN-1 dependent transcriptional response. We constructed a sub-library containing 78 RNAi clones targeting enzymes in catabolic pathways that represent all 20 proteinogenic amino acids and genes involved in glutathione (GSH) synthesis (Table 1). Using this RNAi sub-library, we assessed SKN-1 activity by measuring the fluorescence of animals expressing GFP downstream of the *gst-4* promoter (*gst-4p::GFP*), a well established reporter of SKN-1 (Link and Johnson, 2002). Notably, we found that knockdown of glutathione synthesis, tyrosine or valine catabolism enzymes activate the SKN-1 reporter as previously described, confirming the ability of our screen to identify known regulating enzymes of, and pathways surveilled by, SKN-1 (Figure 1A, Table 1) (Fisher et al., 2008; Wang et al., 2010). Interestingly, we found that genetic perturbation of histidine, glycine and phenylalanine catabolism led to activation of SKN-1 (Figure 1A and Table 1). Together, these results suggest that SKN-1 is a transcription factor downstream of amino acid catabolism which responds to the disruption of these pathways.

**Table 1:** Amino acid catabolism and glutathione synthesis genes screened

RNAi	Score	Source
*H14N18.4*	not scored	Ahringer Library
*haao-1*	not scored	Ahringer Library
*gcs-1*	3	Ahringer Library
*gss-1*	3	Ahringer Library
*fah-1*	3	Ahringer Library
*hach-1*	2	Ahringer Library
*T07 × 10*^*3*^*.3*	2	Ahringer Library
*F30A10.9/stn-1*	1	Ahringer Library
*gldc-1*	1	Ahringer Library
*gsr-1*	1	Ahringer Library
*let-504*	0	Ahringer Library
*gcsh-2*	0	Ahringer Library
*bckd-1B*	0	Ahringer Library
*glna-3*	0	Ahringer Library
*alh-6*	0	Ahringer Library
*T01H3.3*	0	Ahringer Library
*K01C8.1*	0	Ahringer Library
*gstk-1*	0	Ahringer Library
*alh-8*	0	Ahringer Library
*glna-1*	0	Ahringer Library
*uroc-1*	0	Ahringer Library
*T25B9.1*	0	Ahringer Library
*afmd-1*	0	Ahringer Library
*C15B*_*12*_*.8*	0	Ahringer Library
*tdo-2*	0	Ahringer Library
*acdh-6*	0	Ahringer Library
*Y39A1A.3/epg-6*	0	Ahringer Library
*bckd-1A*	0	Ahringer Library
*bckd-1A*	0	Ahringer Library
*qdpr-1*	0	Ahringer Library
*ZK180.5*	0	Ahringer Library
*srbc-64*	0	Ahringer Library
*R102.4*	0	Ahringer Library
*prdh-1*	0	Ahringer Library
*Y7A9A.1*	0	Ahringer Library
*Y79H2A.12/lin-40*	0	Ahringer Library
*T19H12.6*	0	Ahringer Library
*C31H2.4*	0	Ahringer Library
*T21F4.1/T22B7.8*	0	Ahringer Library
*C15B*_*12*_*.1*	0	Ahringer Library
*got-2.2*	0	Ahringer Library
*gcsh-1*	0	Ahringer Library
*cblc-1*	0	Ahringer Library
*gta-1*	−1	Ahringer Library
*ahcy-1*	−3	Ahringer Library
*E01A2.1*	not scored	Vidal Library
*Y45G12C.1*	not scored	Vidal Library
*fah-1*	3	Vidal Library
*gcs-1*	3	Vidal Library
*hgo-1*	2	Vidal Library
*pah-1*	2	Vidal Library
*H14N18.4*	1	Vidal Library
*Y97E10A2.2*	1	Vidal Library
*uroc-1*	1	Vidal Library
*hach-1*	1	Vidal Library
*M04B2.4*	1	Vidal Library
*amdh-1*	1	Vidal Library
*ethe-1*	0	Vidal Library
*T03D8.6*	0	Vidal Library
*gcsh-1*	0	Vidal Library
*B0250.5*	0	Vidal Library
*gst-42*	0	Vidal Library
*haly-1*	0	Vidal Library
*T25B9.1*	0	Vidal Library
*afmd-1*	0	Vidal Library
*qdpr-1*	0	Vidal Library
*got-2.2*	0	Vidal Library
*glna-3*	0	Vidal Library
*B0250.5*	0	Vidal Library
*gcst-1*	0	Vidal Library
*oatr-1*	0	Vidal Library
*K01C8.1*	0	Vidal Library
*acdh-6*	0	Vidal Library
*Y53G8B.1*	0	Vidal Library
*hpd-1*	0	Vidal Library
*afmd-2*	0	Vidal Library
*gcsh-2*	0	Vidal Library
*gst-43*	0	Vidal Library
*got-2.1*	0	Vidal Library
*C53D5.5*	0	Vidal Library
*gstk-1*	0	Vidal Library
*ahcy-1*	−3	Vidal Library

### Knockout of histidine catabolism enzyme AMDH-1 triggers an SKN-1-mediated detoxification response

Our screen revealed that the phenylalanine, glycine and histidine catabolism pathways may be surveilled by SKN-1, and activate this transcription factor when perturbed. We chose the conserved histidine catabolism pathway as a model to further elucidate this mechanism of SKN-1 activation, as histidine is an essential amino acid and its metabolic pathway remains largely uncharacterized in *C. elegans*. *T12A2.1*, which was renamed AMiDoHydrolase domain containing protein 1 (*amdh-1)*, is a conserved amidohydrolase in the histidine catabolism pathway that processes its substrate, 4-imidazolone-5-propanoate (IP), to create N-formimino-L-glutamate (FIGLU) (Figure 1B). To further explore the mechanism of SKN-1 surveillance of amino acid catabolic processes, we used CRISPR-Cas9 to generate a putative null allele, *amdh-1*(*uth29),* by introducing a premature stop codon in exon one of its coding sequence (Figure S1)*.* We found that *amdh-1* mutants robustly induce the SKN-1 reporter strain in an SKN-1-dependent manner, compared to wildtype animals (Figures 1C and 1D). To determine whether *amdh-1* mutation altered global SKN-1 transcription, we analyzed gene expression changes in *amdh-1* mutants versus wildtype animals (Figure 1E and Table S1). We found that a unique subset of SKN-1 targets, the detoxification enzyme family of glutathione-*s*-transferases (GSTs), were among the most upregulated genes in our dataset, suggesting that changing AMDH-1 levels triggers a specific transcriptional output, likely driven by SKN-1. Direct comparison of differentially expressed genes (DEGs) in our dataset to previously published datasets revealed that genes upregulated in *amdh-1* mutants were also highly upregulated in *skn-1(lax188)* gain-of-function animals and downregulated in worms treated with *skn-1* RNAi (Figure 1F) (Nhan et al., 2019; Steinbaugh et al., 2015). In contrast, downregulated genes in *amdh-1* mutant animals were not differentially regulated under *skn-1* loss or gain-of-function, suggesting that AMDH-1 may also impact SKN-1-independent processes. Interestingly, knockdown of *amdh-1* did not affect the expression of another well-characterized SKN-1 reporter (*gcs-1p::GFP*), further confirming that there are distinct transcriptional responses modulated by SKN-1 (Figures S1B and S1C). Taken together, our data show that disruption of histidine catabolism upregulates a specific transcriptional program that is strikingly similar to those activated by SKN-1, and is only a subset of the general oxidative stress response, and is likely tailored to the perturbation of this pathway. This response includes detoxification enzymes but not other known SKN-1 targets such as antioxidant synthesis enzymes.

### SKN-1 activation in *amdh-1* mutants is dependent on known oxidative stress regulators but not canonical p38/MAPK or nutrient signaling pathways

Amino acids play essential roles in maintaining energy homeostasis, a process controlled by a few key nutrient regulators (Efeyan et al., 2015). Therefore, we hypothesized that SKN-1 activation in *amdh-1* mutants may be regulated by these nutrient sensing pathways. To test this hypothesis, we knocked down orthologs of nutrient regulators mTORC1/2 (*let-363)*, RRAGA *(raga-1*), AMPK (*aak-2)*, FOXO (*daf-16*), TFEB (*hlh-30*) and GCN2 (*gcn-2*) and surveyed SKN-1 activation in *amdh-1* mutants. Strikingly, we observed that none of these regulators were completely required for SKN-1 activation (Figures S2A and S2B). Knockdown of *aak-2* had no effect while *daf-16*, *hlh-30* or *raga-1* further increased SKN-1 activation in *amdh-1* mutants. Notably, *gcn-2* or *let-363* knockdown significantly decreased SKN-1 activation in these mutants, although SKN-1 levels remained over two-fold greater (log2(FC) > 1) in *amdh-1* mutants compared to wildtype animals fed RNAi against these genes (Figures S2A and S2B). Interestingly, we observed increased activation of SKN-1 in *raga-1* knockdown conditions, targeting RRAGA/mTORC1, and suppression of activation upon *let-363* knockdown, targeting both mTORC1/mTORC2. These data suggest that activation of SKN-1 in *amdh-1* mutants may partially depend on GCN2 and mTORC2 while it is independent of mTORC1/RRAGA and other nutrient regulators such as AMPK, FOXO and TFEB.

**Figure 2:**
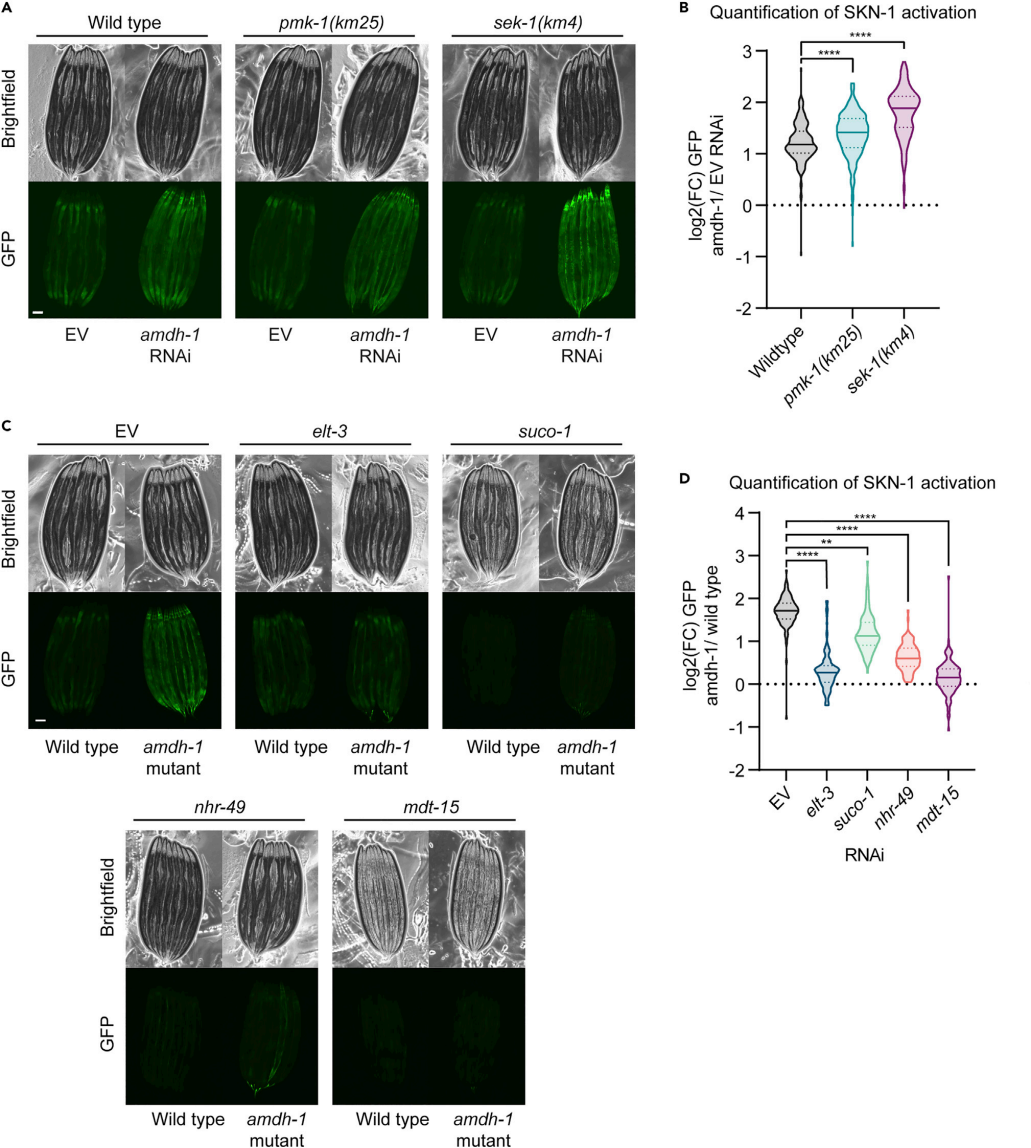
Genetic requirements of SKN-1 activation upon perturbation of histidine catabolism (A) Fluorescent images of SKN-1 reporter animals in a wildtype, *sek-1(km4)* or *pmk-1(km25)* mutant animals fed *amdh-1* RNAi. Scale bar, 100 μm. (B) Quantification of SKN-1 activation (*amdh-1* RNAi normalized to median of EV) from (A), Data shown are representative of n = 3 biological replicates with n > 172 animals per condition for each replicate. ∗∗∗∗ = p < 0.0001 using a one-way ANOVA. (C) Fluorescent images of SKN-1 reporter animals in a wildtype or *amdh-1(uth29)* mutant background fed RNAi targeting *elt-3*, *suco-1, nhr-49,* and *mdt-15*. Scale bar, 100 μm. (D) Quantification of SKN-1 activation (*amdh-1* mutant normalized to median of wild type) from (C), Data shown are representative of n = 3 biological replicates with n > 88 animals per condition for each replicate. ∗∗ = p < 0.01, ∗∗∗∗ = p < 0.0001 using a one-way ANOVA.

In *C. elegans*, SKN-1 can be activated via phosphorylation by the p38/MAPK ortholog, PMK-1, in a signaling cascade that requires the MAPKK, SEK-1 (Inoue et al., 2005). To assess the requirements of these well established regulators on SKN-1 activation, we tested whether animals with mutations in this pathway can still activate SKN-1 upon *amdh-1* knockdown. We observed that *pmk-1(km25)* and *sek-1(km4)* mutants fail to suppress SKN-1 activation and instead exhibit increased activation with *amdh-1* knockdown (Figures 2A and 2B). These data show that canonical MAPK regulators are not required for SKN-1 activation in the face of metabolic perturbations and may even negatively regulate this response.

To identify genetic pathways required for SKN-1 activation in *amdh-1* mutants, we performed a genetic suppressor screen using EMS mutagenesis on SKN-1 reporter animals in an *amdh-1(uth29)* background. Using a combination of backcrossing and deep sequencing as previously described (Lehrbach et al., 2017), we identified six alleles in four genes that are required for SKN-1 activation in *amdh-1* mutants (Figure S2C). Among the mutants identified was a putative DNA binding domain mutant of *skn-1* that is present across all four isoforms (Figure S2C). These worms are slow growing, likely due to the requirement for SKN-1 in development (Bowerman et al., 1992). The discovery of this SKN-1 allele validates the screen and supports the previous finding that this phenotype is dependent on *skn-1* (Figures 1C and 1D). Among the remaining mutations found in our screen, we identified a previously undiscovered SKN-1 regulator, *suco-1,* and a previously identified regulator, *elt-3*, as suppressors of SKN-1 activity in *amdh-1(uth29)* mutant animals (Figure S2C). RNAi knockdown of *suco-1* or *elt-3* suppress SKN-1 activation in *amdh-1* mutants, phenocopying the EMS mutants and suggesting a dependence of these regulators on SKN-1 activation (Figures 2C and 2D). *suco-1* encodes an ortholog of the SLP/EMP65 complex identified in yeast to function in ER protein homeostasis, a process previously implicated in the OxSR (Glover-Cutter et al., 2013; Zhang et al., 2017). *elt-3* is a required factor for induction of OxSR gene expression (Hu et al., 2017). The existing role of *elt-3* in the OxSR prompted further investigation of other required nuclear factors.

SKN-1 drives expression of detoxification enzymes, such as GST-4, under conditions of oxidative stress with the help of nuclear factors like ELT-3, nuclear hormone receptor NHR-49, and mediator complex subunit, MDT-15 (Goh et al., 2014, 2018; Hu et al., 2017; Wu et al., 2016). As these nuclear factors are well established to be required for both stress response gene transcription and survival upon oxidative stress, we hypothesized that they may also be required for SKN-1 activation in *amdh-1* mutants. We found that knockdown of *nhr-49* or *mdt-15* significantly suppressed the activation of SKN-1 in *amdh-1(uth29)* mutants (Figures 2C and 2D). Together, these data suggest that activation of SKN-1 in animals with perturbed histidine catabolism is dependent on canonical nuclear regulators of the OxSR.

### Enzymes HALY-1 and Y51H4A.7/UROC-1 are required for SKN-1 activation in AMDH-1 mutants

**Figure 3:**
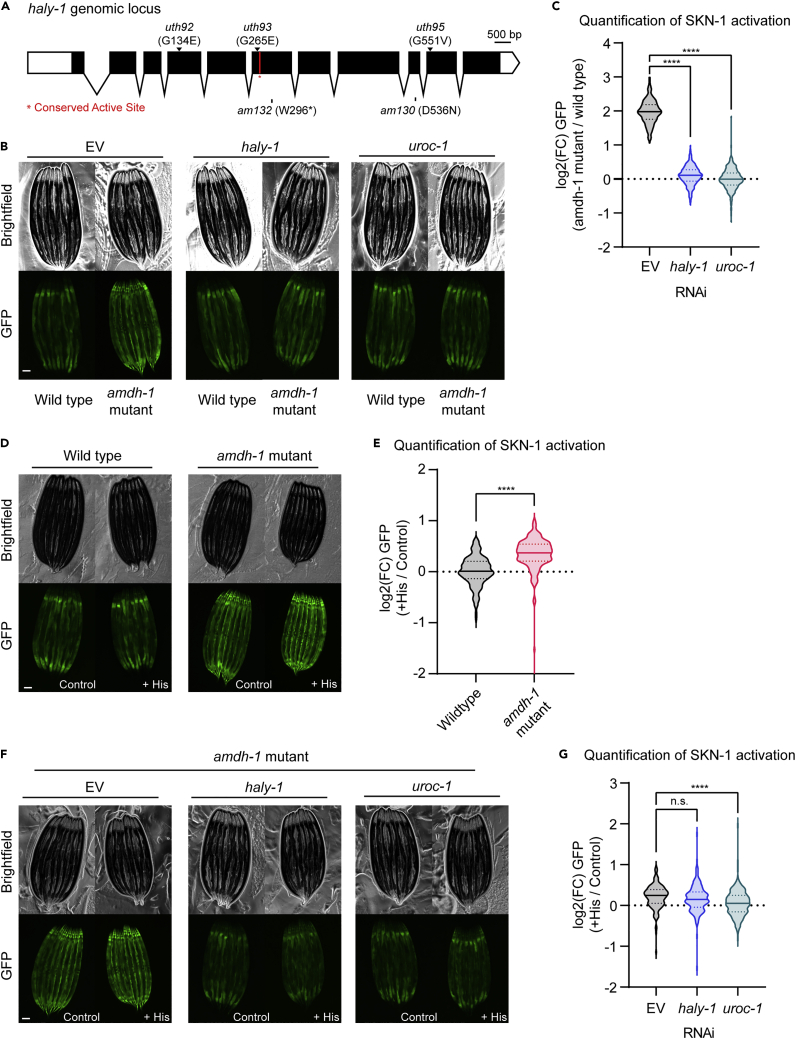
Activation of SKN-1 requires upstream histidine catabolism enzymes and likely proceeds through the buildup of a metabolic intermediate (A) Schematic of *haly-1* genomic locus labeling novel alleles from EMS screen (top, arrows). A conserved active site is labeled in red and two existing alleles are labeled (bottom). Scale bar, 500 bp. (B) Fluorescent images of SKN-1 reporter animals in a wildtype or *amdh-1(uth29)* mutant background fed RNAi targeting *haly-1* and *uroc-1*. Scale bar, 100 μm. (C) Quantification of SKN-1 activation (*amdh-1* mutant normalized to median of wild type) from (B), Data shown are representative of n = 3 biological replicates with n > 250 animals per condition for each replicate. ∗∗∗∗ = p < 0.0001 using a one-way ANOVA. (D) Fluorescent images of SKN-1 reporter animals in a wildtype or *amdh-1(uth29)* mutant background with (+His) or without (control) 10mM histidine added to the media. Scale bar, 100 μm. (E) Quantification of SKN-1 activation (*+*His normalized to median of control) from (D), Data shown are representative of n = 3 biological replicates with n > 141 animals per condition for each replicate. ∗∗∗∗ = p < 0.0001 Mann-whitney U test. (F) Fluorescent images of SKN-1 reporter animals in an *amdh-1(uth29)* mutant background fed RNAi with or without 10mM histidine added to the media. Scale bar, 100 μm. (G) Quantification of SKN-1 activation (*+*His normalized to median of control) from (F), Data shown are representative of n = 3 biological replicates with n > 193 animals per condition for each replicate. ∗∗∗∗ = p < 0.0001 one-way ANOVA.

Through the EMS suppressor screen, we also identified three independent alleles of *haly-1 (uth92, uth93, uth95)*, the conserved histidine ammonia lyase that is rate limiting for the histidine catabolism pathway, as suppressors of SKN-1 activity (Figures 3A and S2C). Expression of a wildtype copy of *haly-1* rescued the suppression of SKN-1 activation in *haly-1(uth92)* and *haly-1(uth93)* mutants, confirming the causative nature of these mutations (Figures S3A and S3B).

AMDH-1 is the third enzyme in the catabolism of histidine to glutamate. Two upstream enzymes conserved in *C. elegans*, HALY-1 and Y51H4A.7*,* renamed to UROCanate hydratase protein 1, UROC-1, catalyze histidine to glutamate conversion through the formation of two intermediate catabolites, urocanate and IP (Figure 1B). One possible mechanism, supported by the finding that *haly-1* mutants suppress SKN-1 activation in *amdh-1* mutants, is that catabolite buildup activates SKN-1. To explore this possibility, we performed epistasis experiments using RNAi to knock down the upstream enzymes of the histidine catabolism pathways in *amdh-1* mutant animals. If a buildup of the second catabolite, IP, leads to SKN-1 activation, knockdown of *uroc-1* would also suppress SKN-1 activation. We found that RNAi knockdown of *haly-1* phenocopies the suppression seen in *haly-1* mutants and that *uroc-1* completely suppressed the activation of SKN-1 in *amdh-1* mutants (Figures 3B and 3C). These findings support the hypothesis that SKN-1 activation in these mutants proceed through a catabolite intermediate, likely IP.

### Histidine supplementation amplifies SKN-1 activation in AMDH-1 mutants

The poor solubility and short half-life of catabolites upstream of *amdh-1*, urocanate and IP, prevented the direct testing of catabolite activation. Rather, we tested whether increasing flux through the histidine catabolism pathway differentially affects *amdh-1* mutants compared to wild type animals. Notably, this differential activation would depend on one or both of the upstream enzymes, *haly-1* and *uroc-1*, if the mechanism proceeds through a catabolite intermediate. Accordingly, we supplemented SKN-1 reporter animals, in both wildtype and *amdh-1* mutant backgrounds, with histidine. Strikingly, *amdh-1* mutant animals exhibit a robust activation of SKN-1 upon histidine supplementation when compared to wildtype animals (Figures 3D and 3E). Knockdown of *haly-1* resulted in a very mild decrease of SKN-1 activation in *amdh-1* mutants supplemented with excess histidine, although this decrease was not statistically significant in all replicates. However, knockdown of *uroc-1* completely suppresses SKN-1 activation (Figures 3F and 3G). These data further support a model that the catabolite intermediate IP drives SKN-1 activation, although the direct nature of this activation remains unclear.

### *amdh-1* mutants are sensitive to heat stress and resistant to oxidative stress

**Figure 4:**
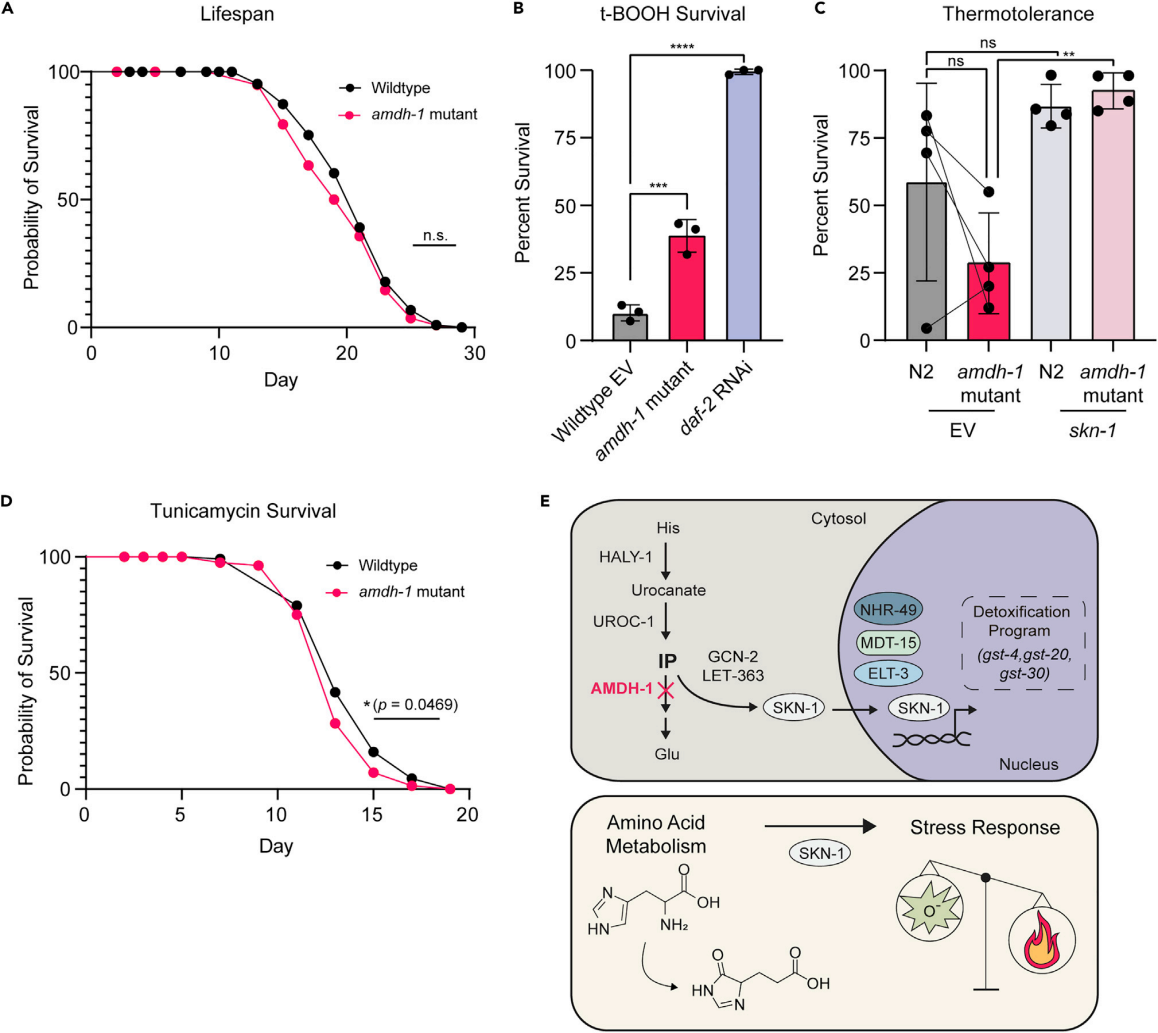
Physiological consequences of SKN-1 activation in *amdh-1* mutants (A) Survival of wildtype animals (N2) and *amdh-1(uth29)* mutant worms at 20°C. Each data point represents one biological replicate of n > 40. One-way ANOVA with Šídák’s multiple comparisons test, ∗∗ = p < 0.01. (B) Survival of animals on plates containing 7.5mM t-BOOH for 16 h. One-way ANOVA with Šídák’s multiple comparisons test, ∗∗∗ = p < 0.001, ∗∗∗∗ = p < 0.0001. (C) Thermotolerance of animals shifted to heat shock temperature (34°C) for 14–15 h. Solid lines connecting points represent replicates complete on the same day to show the relative trend of thermotolerance between wild type and mutant animals. One-way ANOVA with Šídák’s multiple comparisons test, ∗∗ = p < 0.01. (D) Survival of animals on 25 ng/μL tunicamycin plates. (E) Schematic of findings. *Top* - Perturbation of histidine catabolism via *amdh-1* mutation leads to a buildup of a catabolite that, directly or indirectly, activates a transcriptional program of detoxification enzymes driven by SKN-1. This response requires nuclear factors NHR-49, MDT-15 and ELT-3. *Bottom* - Activation of SKN-1 via perturbation of histidine catabolism leads to a physiological tradeoff of increased oxidative stress resistance and decreased heat tolerance.

SKN-1 can be either beneficial or detrimental to organismal physiology and aging depending on expression level. For example, moderate activation of SKN-1 extends lifespan while high expression can shorten lifespan (Paek et al., 2012; Tullet et al., 2008). Moreover, activation of SKN-1 increases oxidative stress survival while decreasing survival to other stressors such as heat (Crombie et al., 2016; Deng et al., 2020). Considering the strong induction of SKN-1 upon *amdh-1* loss of function, we questioned whether this activation was beneficial or detrimental to organismal health. First, we assessed the lifespans of *amdh-1(uth29)* mutants compared to wildtype animals and found no significant effect on adult lifespan (Figure 4A). We next tested whether SKN-1 activation affects the animal’s ability to survive different stress conditions. Predictably, *amdh-1* mutants are resistant to *tert*-butyl hydroperoxide, an organic peroxide known to induce the OxSR, likely representing a “priming” effect that SKN-1 has on these animals to survive oxidative stress (Figure 4B). Surprisingly, we observed a consistent decrease in resistance to thermal stress across replicates, and an average of 50% decrease in thermotolerance compared to wildtype animals. The decreased thermotolerance of *amdh-1* mutants was completely rescued by RNAi knockdown of *skn-1*, suggesting that SKN-1 activation is detrimental to thermotolerance (Figure 4C). Additionally, stress survival on tunicamycin was also modestly reduced (Figure 4D). Notably, we did not observe a change in any other fluorescent reporters that measure the activation of other cellular stress responses (Figures S4A–S4E). Together, our data suggest that SKN-1 activation in *amdh-1* mutants drives physiological changes that promote survival under oxidative stress conditions but are detrimental to heat and ER stress resilience without a significant impact on these well defined transcriptional stress responses.

## Discussion

### SKN-1 as an effector downstream of amino acid catabolism pathways

Previous studies have reported that perturbation of tryptophan, threonine, or proline catabolism evoke an SKN-1-mediated transcriptional response (Fisher et al., 2008; Pang et al., 2014; Tang and Pang, 2016; Ravichandran et al., 2018). Our work expands this list to include glycine, valine, phenylalanine and histidine catabolism as pathways that may be surveilled by SKN-1, which can have direct effects on stress resilience. Further, we demonstrate that knockdown of a conserved amidohydrolase in the histidine catabolism pathway leads to a buildup of a catabolite, likely IP, to activate a transcriptional response driven by SKN-1. This response appears partially dependent on nutrient regulators, GCN-2 and mTORC2, and OxSR regulators ELT-3, NHR-49 and MDT-15 (Figure 4E). These data suggest that GCN-2 and mTORC are involved in SKN-1 activation, which is unsurprising considering their roles as central metabolic regulators. Finally, the activation of SKN-1 results in differential effects on stress resistance with an increased resilience to oxidative stress and a mild sensitivity to heat stress.

While it is unknown whether SKN-1 activation in this context is a direct or indirect consequence of catabolite buildup, existing literature suggests that multiple amino acid catabolites can signal to SKN-1. For example, proline catabolism is modulated upon pathogen exposure to accumulate the intermediate pyrroline-5-carboxylate (P5C) to activate SKN-1 through ROS production (Tang and Pang, 2016). Other reports have demonstrated that disruption of tyrosine catabolism, through mutation in fumarylacetoacetate hydrolase, *fah-1*, causes stunted growth and intestinal degradation through tyrosine catabolites (Fisher et al., 2008). Intriguingly, *fah-1* mutants have increased SKN-1 activity (Fisher et al., 2008). Our findings help unify these previously observed phenomena and suggest that SKN-1 may integrate information from multiple catabolite activators into transcriptional programs to affect physiology.

Interestingly, in response to mutation of *amdh-1*, there is an upregulation of a subset of target genes including *gst-4*, but not another well known SKN-1 target, *gcs-1*. Previous studies have reported such discordance between SKN-1 activation and the expression of these specific genes. For example, in an RNAi screen for genes that activate a *gcs-1* reporter when knocked down, Wang and colleagues found that a small subset of them failed to also activate expression of *gst-4* (Wang et al., 2010)*.* Furthermore, SKN-1 has been shown to upregulate specific subsets of genes depending on the stress encountered, demonstrating that the regulation of SKN-1 is complex and likely represents the ability of this transcription factor to regulate tailored responses subject to the nature of the perturbation (Oliveira et al., 2009). Our results fit within these observations and add to the list of many perturbations that evoke an SKN-1 mediated response.

Importantly, amino catabolism pathways are implicated in disease progression and cancer treatment. A previous study has shown that disruption of this pathway decreases sensitivity to the chemotherapeutic methotrexate and increasing flux through this pathway has been proposed to increase methotrexate efficacy in patients (Kanarek et al., 2018). While simple dietary intervention is appealing, our understanding of the consequences of catabolite buildup remains incomplete. Indeed, one study indicates that histidine supplementation can cause hepatic enlargement in patients with liver disease (Holeček, 2020). Moreover, in type I tyrosinemia, tyrosine catabolite buildup is thought to be a main source of damage to proteins and DNA, contributing to pathology (Ferguson et al., 2010). We observe that histidine catabolites may also signal to effectors, such as SKN-1, to modulate physiology. Thus, further studies to uncover the clear role of catabolite intermediates as important modulators of cell signaling is at the forefront of understanding human disease.

### 4-Imidazolone-5-propanoate (IP) as a signaling catabolite

Enzymes of the histidine catabolism pathway are highly conserved from bacteria to humans (Bender, 2012). Interestingly, mutations in the conserved IPase in *Klebisella aerogenes* are innocuous unless supplemental histidine is added and upstream enzymes are active, in which case the bacteria are poisoned and fail to grow (Boylan and Bender, 1984). This phenotype bears striking resemblance to the phenotype observed here, in which *C. elegans* mutants for the IPase, *amdh-1,* show exaggerated phenotypes when supplemented with histidine, dependent on the upstream enzymes *haly-1* and *uroc-1*. To date, no direct experimentation has shown the toxicity of this catabolite, likely due to the short half-life of IP (Bowser Revel and Magasanik, 1958; Rao and Greenberg, 1961). IP may either directly or indirectly activate SKN-1 through a breakdown product, production of oxidative stress or through a distinct mechanism. Indeed, 4-imidazolones have previously been shown to cause oxidative stress and make up many advanced glycation end-products (AGEs), biomarkers that correlate with aging and metabolic disease (Niwa et al., 1997; Omar et al., 2018).

### Requirements of SKN-1 activation in *amdh-1* mutants

To date, the MAPK signaling pathway has been reported to control nearly all instances of SKN-1 activation in *C. elegans* (Blackwell et al., 2015). Here we report an SKN-1 transcriptional response to altered histidine catabolism that is independent of conserved MAPK components *sek-1* and *pmk-1*. Although the identity of upstream signaling components remain elusive, several kinases known to influence SKN-1 activation remain as candidates downstream of the signaling catabolite (Kell et al., 2007). Notably, we found that knockdown of *gcn-2* partially suppressed SKN-1 activation in *amdh-1* mutants. *gcn-2* is a conserved protein kinase which functions in the integrated stress response (ISR) (Pakos-Zebrucka et al., 2016). Interestingly, the homolog of SKN-1, NRF2, has known functions in regulating the transcription of ATF4, the core effector of the ISR, in mammals (Pakos-Zebrucka et al., 2016). Moreover the *gcn-2* inhibitor, *impt-1,* extends the lifespan of *C. elegans* and requires SKN-1, highlighting potential interactions between *gcn-2*, the ISR and SKN-1. However, we do not see a significant expression change of the ATF4 homolog, *atf-5*, or its putative targets in *amdh-1* mutants (Table S1) (Statzer et al., 2022). Further studies are needed to determine the entirety of the signaling cascade that culminates in the activation of SKN-1 upon metabolic perturbations, as the partial dependence of *gcn-2* suggests other factors are involved. Interestingly, we identified *suco-1* as a suppressor of SKN-1 activation in *amdh-1* mutants. *suco-1* is a homolog of the SLP1 protein in yeast, which is hypothesized to participate in protecting nascent proteins from degradation during folding in the ER (Zhang et al., 2017)*.* Previous work has identified UPR^ER^ components such as *ire-1* and *hsp-4* in the transcriptional response to oxidants arsenite and tBOOH (Glover-Cutter et al., 2013). If *suco-1* functions in a similar capacity in *C. elegans* as it does in yeast, this could implicate other ER protein homeostasis pathways in the regulation of SKN-1. Indeed, an ER-associated isoform of SKN-1, SKN-1A, is known to be a monitor of proteasome function and may modulate crosstalk between the ER and SKN-1 (Lehrbach et al., 2017).

### SKN-1 activation differentially affects stress resilience

Titration of SKN-1 expression is important for the pro-longevity nature of this transcription factor. Moderate overexpression of SKN-1 or mutation of the negative regulator *wdr-23* extends the lifespan of *C. elegans,* while hypomorphic mutants or worms treated with *skn-1* RNAi exhibit a shortened lifespan (Grushko et al., 2021; Ganner et al., 2019; Tullet et al., 2008, 2017; Tang and Choe, 2015). However, gain-of-function animals with constitutive expression of SKN-1 and animals containing high-copy arrays of SKN-1 exhibit a mild decrease in lifespan (Paek et al., 2012; Tullet et al., 2008). Additionally, activation of SKN-1 is beneficial for oxidative stress survival while detrimental to survival under other conditions such as heat, ER or mitochondrial stress (Deng et al., 2020). SKN-1 activation upon metabolic perturbation, as shown here, provides short term benefit to survive oxidative stress but comes at the cost of sensitivity to heat and ER stress. This may represent a physiological trade off, where SKN-1 prioritizes the allocation of cellular resources to defend against a perceived threat at the cost of sensitivity to other perturbations. Identification and study of the physiologically relevant consequences of SKN-1 activation will be crucial to understanding how modulation of this master transcription factor may be leveraged to affect human disease.

### Limitations of the study

This study describes a role for SKN-1 downstream of the histidine catabolism pathway through the signaling of a metabolic intermediate, IP. This work, and others (Fisher et al., 2008; Ravichandran et al., 2018; Tang and Pang, 2016), suggests that SKN-1 can respond to buildup of metabolic intermediates to integrate information, perhaps about metabolic state, and influence physiology. Whether the activation of SKN-1 is a result of a direct interaction with these metabolites or through a more indirect mechanism, perhaps via formation of various stressors known to activate SKN-1, is unclear. Additionally, though our sub-library screen results implicate SKN-1 downstream of tyrosine, valine, glycine, phenylalanine and histidine catabolism, the limited scope of screened genes limits the discovery of other upstream metabolic pathways. Further work is necessary to comprehensively catalog the metabolic pathways that signal to SKN-1. Finally, we recognize that some decreases in the data are not statistically significant and thus should be interpreted carefully.

## STAR★Methods

### Key resources table

**Table:** 

REAGENT or RESOURCE	SOURCE	IDENTIFIER
**Bacterial and virus strains**		

OP50	CGC	N/A
HT115	CGC	N/A

**Chemicals, peptides, and recombinant proteins**		

Agarose, low melting	Sigma-Aldrich	A9414-10G
Bacto Peptone	Fisher Scientific	DF0118072
BD Difco granulated agar	VWR	90000-782
Calcium chloride dihydrate	VWR	97061-904
Carbenicillin	BioPioneer	C0051-25
Chloroform	Sigma-Aldrich	34854
Cholesterol	Sigma-Aldrich	57-88-5
IPTG dioxane free	Denville Scientific	CI8280-4
Isopropanol	Fisher Scientific	AC327272500
LB Broth Miller	Fisher Scientific	BP1426500
Magnesium sulfate heptahydrate	VWR	EM-MX0070-3
Potassium Hydroxide	Fisher Scientific	P250-500
Potassium phosphate dibasic	VWR	EM-PX1570-2
Potassium phosphate monobasic	VWR	EM-PX1565-5
Sodium Azide	Sigma-Aldrich	S2002-25G
Sodium Chloride	EMD Millipore	SX0420-5
Sodium hypochlorite solution	Ricca Chemical	R74957000-4B
Sodium phosphate dibasic	VWR	71003-472
Tetracycline hydrochloride	Sigma-Aldrich	T7660-5G
Trizol	Fisher Scientific	15596018
Tunicamycin	Millipore	654380
L-Histidine	Sigma-Aldrich	H8000-100G
Luperox TBH70X, tert-Butyl hydroperoxide solution	Sigma-Aldrich	458139-25ML

**Critical commercial assays**		

KAPA mRNA HyperPrep Kit	Roche	KK8581
KAPA SI Adapter Kit	Roche	KK8700
QIAquick PCR Purification Kit	Qiagen	28106
QIAprep Spin Miniprep Kit	Qiagen	27106
RNeasy Mini Kit	Qiagen	74106

**Deposited data**		

RNA-seq; *amdh-1(uth29)* vs. wildtype)	This study	SRA accession PRJNA801069
RNA-seq; *skn-1(lax188)* vs. wildtype)	Nhan et al., 2019	GEO accession GSE123531
RNA-seq; *skn-1* RNAi vs. control)	Steinbaugh et al., 2015	GEO accession GSE63075

**Experimental models: Organisms/strains**		

*C. elegans*: strain N2 (Bristol)	CGC	N2
*C. elegans*: strain LD1171: ldIs3 [gcs-1p::GFP + rol-6(su1006)]	CGC	LD1171
*C. elegans*: strain CL2166: dvIs19[pAG15(gst-4p::GFP::NLS)] III	CGC	CL2166
*C. elegans*: strain *AM446: rmIs223[pC12C8.1::GFP; rol-6(su1006) II]*	Morley and Morimoto, 2004	AM446
*C. elegans*: strain AGD3126: zcIs13[hsp-6p::GFP]	This paper	AGD3126
*C. elegans*: strain AGD3111: dvIs19[pAG15(gst-4p::GFP::NLS)] III; sek-1(km4) X	This paper	AGD3111
*C. elegans*: strain AGD3013: uth93 ;*amdh-1*(uth29)III; dvIs19[pAG15(gst-4p::GFP::NLS)]; uthEx967[haly-1p::haly-1::haly-1 3′ UTR, myo-3p::mCherry]	This paper	AGD3013
*C. elegans*: strain AGD2530: uth93;*amdh-1*(uth29)III; dvIs19[pAG15(gst-4p::GFP::NLS)]	This paper	AGD2530
*C. elegans*: strain AGD2529: uth92;*amdh-1*(uth29)III; dvIs19[pAG15(gst-4p::GFP::NLS)]	This paper	AGD2529
*C. elegans: strain* AGD2432: pmk-1(km25) IV; dvIs19[pAF15(gst-4p::GFP::NLS)]	This paper	AGD2432
*C. elegans: strain AGD2307:amdh-1(uth29)III; dvIs19[pAG15(gst-4p::GFP::NLS)]*	This paper	AGD2307
*C. elegans: strain AGD2306: amdh-1(uth29)III; rmIs223[pC12C8.1::GFP; rol-6(su1006) II]*	This paper	AGD2306
*C. elegans*: strain AGD2053: *zcls4[hsp-4p::GFP]V* (SJ4005 5× backcross)	This paper	AGD2053
*C. elegans: strain AGD1848: amdh-1(uth29)III*	This paper	AGD1848

**Software and algorithms**		

ECHO Software (Revolve Scope)	ECHO	N/A
COPAS Software	Union Biometrica, Inc.	N/A
GraphPad Prism	GraphPad	Version 8.0

### Resource availability

#### Lead contact

Further information and requests for resources and reagents should be directed to and will be fulfilled by the lead contact, Dr. Andrew Dillin (dillin@berkeley.edu).

#### Materials availability

Any requests for *C. elegans* strains original to this work should be directed to, and will be fulfilled by, the lead contact.

### Experimental model and subject details

#### *C. elegans* maintenance

All *C. elegans* strains were maintained at 15°C on NGM plates with OP50 *E. coli* B strain. All experiments were performed at 20°C on RNAi plates (NGM agar, 1 mM IPTG, 100 μg/mL carbenicillin) with HT115 *E. coli* K12 strain bacteria containing the RNAi plasmid pL4440 empty vector as a negative control (EV) or containing sequence to synthesize a double-stranded RNA against a target gene unless otherwise stated. All RNAi constructs were isolated from the Vidal or Ahringer RNAi library and sequence verified before using. For all experiments, eggs were obtained using a standard bleaching protocol (1.8% sodium hypochlorite and 0.375 M KOH) and arrested at the L1 stage overnight in M9 (22 mM KH_2_PO_4_ monobasic, 42.3 mM Na_2_HPO_4_, 85.6mM NaCl, 1 mM MgSO_4_) without food for synchronization. The next day, synchronized L1 animals were placed on HT115 bacteria and grown until day 1 of adulthood. For histidine supplementation experiments, plates were supplemented with 10mM histidine that was buffered with HCl to pH 7.0. Animals were grown on histidine supplemented plates after L1 synchronization for the duration of the entire experiment.

*haly-1* rescue experiments were performed using a ∼4.3kb amplicon from the genomic DNA of N2 (bristol) animals to include a putative promoter region (∼1.1 kb) and complete CDS (∼2.6kb, including introns) flanked on each side by the endogenous 5′ and-3′ UTRs as annotated by wormbase (∼0.3kb each). This amplicon was generated in a standard PCR reaction using N2 genomic DNA with the forward primer oPF388 (5′-ttgtccaataaacctttgtcc-3′) and reverse primer oPF389 (5′-tccatataaccctgtaactcc -3′) and sequenced verified using standard Sanger sequencing after PCR purification. Array positive animals were generated by injecting *haly-1(uth92)* or *haly-1(uth93)* animals with purified amplicon at 40 ng/μL along with a co-injection marker (myo-3p::mCherry) at 5 ng/μL. Two independent arrays were isolated from different parent animals for each *haly-1* mutant allele.

### Method details

#### Lifespan and stress assays

Lifespan measurements were assessed on RNAi plates (standard NGM agar supplemented with 1mM IPTG and 100ug/mL carbenicillin) with HT115 bacteria carrying pL4440 empty vector RNAi. Worms were synchronized by standard bleaching/L1 arresting as described and kept at 20°C throughout the duration of the experiment. Adult worms were moved away from progeny onto fresh plates for the first 5–7 days until progeny were no longer visible and scored every 1 to 2 days until all animals were scored. Animals with bagging, vulval explosions, or other age-unrelated deaths were censored and removed from quantification. For tunicamycin survival assays, animals were moved onto tunicamycin (25 ng/μL) or 1% DMSO plates at D1 of adulthood and scored as described for standard lifespan measurements. For thermotolerance, worms were synchronized by bleaching as described above, L1 arrested, and plated on RNAi plates with HT115 bacteria carrying pL4440 empty vector or other RNAi. At D1, 15–20 worms per plate with 3-4 plates per condition were exposed to 34°C heat via incubator for 14–15 hours. Plates were then removed from the incubator and manually assessed for movement and pharyngeal pumping, using light head taps where necessary, to determine survival. Worms that displayed internal hatching (bagging) or crawled onto the side of the plate and desiccated were censored and omitted from the final analysis. Percent alive was calculated using the number of living worms divided by the total number of worms excluding censored animals for each strain. For oxidative stress survival, worms were bleach synchronized, L1 arrested, and plated on RNAi plates with HT115 bacteria carrying empty vector or *daf-2* RNAi. At D1, ∼100 animals per condition were transferred to 4-5 NGM plates containing 7.5mM t-booh (Luperox TBH70X, Sigma). Worms were scored for survival every 2 hours, starting at 12 hours, until the 16 hour time point. Survival statistics for each replicate of lifespan and stress assays can be found in Table S2.

#### Fluorescence imaging, quantification and representation

Image acquisition was performed as previously described (Bar-Ziv et al., 2020). Briefly, day 1 animals were picked under a standard dissection microscope onto a solid NGM plate that contained a ∼15μL drop of 100 nM sodium azide. Immobilized worms were aligned head to tail and images were captured on an Echo Revolve R4 microscope equipped with an Olympus 4× Plan Fluorite NA 0.13 objective lens, a standard Olympus FITC filter (e×470/40; em 525/50; DM 560), and an iPad Pro for the camera and to drive the ECHO software.

To quantify fluorescence, a COPAS large particle biosorter was used as previously described (Bar-Ziv et al., 2020). Data were collected gating for size (time of flight [TOF] and extinction) to exclude eggs and most L1 animals. Data were processed by censoring events that reached the maximum peak height for Green or Extinction measurements (PH Green, PH Ext = 65532) and censoring events < 300 TOF to exclude any remaining L1 animals. For the reporters with low basal fluorescence (AGD2053, AGD3126), data > 0 were included. For reporter strains with visible basal fluorescence (CL2166, AGD2307), data ≥ 10 were included for subsequent statistical analysis. All fluorescence data were normalized to TOF to account for worm size. For all *amdh-1(uth29)* mutant experiments, ‘SKN-1 activation’ was quantified by normalizing Green/TOF value for each mutant to the median of the wildtype population for each condition. For the MAPK mutant experiments (Figure 2A), ‘SKN-1 activation’ was quantified by normalizing the Green/TOF value for each mutant fed *amdh-1* RNAi to the median of that mutant fed EV RNAi. All fold changes were log2 transformed and plotted as violin plots using GraphPad Prism. Solid lines on the violin plot represent the log2 transformed median value, while the dotted lines above and below the median represent the 1st and 3rd quartiles, log2 transformed, respectively.

#### RNA isolation, sequencing, and analysis

Animals were bleach synchronized and grown to Day1 adulthood on empty vector RNAi plates. At least 2,000 animals per condition per replicate were washed off plates using M9 and collected. After a 30 second spin down at 1,000 RCF, M9 was aspirated, replaced with 1mL Trizol, and the tube was immediately frozen in liquid nitrogen to be stored at −80°C for downstream processing. RNA was harvested after 3 freeze thaw cycles in liquid nitrogen/37°C water bath. After the final thaw, 200μL of chloroform were added to the sample, vortexed, and the aqueous phase was collected after centrifugation in a gel phase lock tube. RNA was isolated from the obtained aqueous phase using a Qiagen RNeasy MiniKit according to manufacturer’s directions. Library preparation was performed using Kapa Biosystems mRNA Hyper Prep Kit (Roche, product number KK8581) using dual index adapters (KAPA, product number KK8722). Sequencing was performed using Illumina HS4000, mode SR100, through the Vincent J. Coates Genomic Sequencing Core at University of California, Berkeley.

For RNA-seq analysis of *amdh-1(uth29)* mutants, the raw sequencing data were uploaded to the Galaxy project web platform and the public server at usegalaxy.org was used to analyze the data (Afgan et al., 2016). Paired end reads were aligned using the Kallisto quant tool (Version 0.46.0) with WBcel235 as the reference genome. Fold changes and statistics were generated using the DESeq2 tool with Kallisto quant count files as the input. Volcano plots were generated using the GraphPad Prism version 8.0.0 for Windows (GraphPad Software, San Diego, California USA, www.graphpad.com) on the fold change and adjusted-p values generated by the DESeq2 analysis (Table S1). For analysis of previously published data, raw reads were downloaded from the Gene Expression Omnibus (GEO), (Accession: GSE123531 and GSE63075) and analyzed as described above.

#### EMS mutagenesis screen to find suppressors of SKN-1 activation

*amdh-1(uth29); gst-4p::GFP::NLS* (strain AGD2307) were mutagenized to find suppressors of *gst-4p::GFP* signal. Briefly, ∼150 L4 animals were picked into a 1.5mL eppendorf tube containing 1mL M9 buffer and spun down at 1,000 RPM for 1 minute. The M9 was aspirated from the tube and replaced with 1mL fresh M9 and spun again. To the washed worm pellet, 5μL of EMS was added, the tube was parafilmed and left nutating at 20C for 4 hours. After incubation, the worms were spun down and rinsed 4 times with 1mL M9. Waste was collected and neutralized with 1:1 KOH before discarding. Rinsed, mutagenized, worms were plated overnight. The next day, mutagenized worms were picked onto 10 large plates seeded with OP50 bacteria, 10 per plate, and allowed to lay eggs for 24 hours. Three days later, adult F1 animals were bleached and plated onto fresh large plates. F2 mutants were screened for suppression of *gst-4p::GFP* under a fluorescent microscope compared to age matched, un-mutagenized, conrol animals.

Genomic DNA was extracted from mutants of interest using the Puregene Cell and Tissue Kit (Qiagen), as previously described (Lehrbach et al., 2017). 2ug of purified DNA was sheared using a Covaris S220 focused-ultrasonicator to produce ∼400 bp fragments. Library preparation was performed with 1ug of sheared DNA using Kapa Biosystems Hyper Prep Kit (Roche, product number KK8504) dual index adapters (KAPA, product number KK8727). Sequencing was performed using the Illumina NovaSeq6000 platform through the Vincent J. Coates Genomic Sequencing Core at University of California, Berkeley. Raw reads were uploaded to the Galaxy project web platform and the public server at usegalaxy.org was used to analyze the data (Afgan et al., 2016). Reads were aligned using the Bowtie2 tool with WBcel235/ce11 as the reference genome. The MiModD tool suite (Baumeister lab) was used on the Variant Allele Contrast (VAC) mapping mode to call, extract and filter variants to compare mutants to the parental, un-mutagenized strain. Causative genes were identified through a combination of genetic complementation, deep sequencing, and RNAi phenocopy experiments.

### Quantification and statistical analysis

All statistical analyses are specifically described in figure legends and in the Experimental methods above. Sample sizes are reported in the figure legend where *n =* animals. GraphPad Prism software version 8.0.0 for Windows (GraphPad Software, San Diego, California USA, www.graphpad.com) was used for all statistical tests and graphical representations.

## Data Availability

The raw RNA-seq data were uploaded to the NCBI short read archive (SRA) and are publicly available as of the date of publication. Accession numbers are listed in the Key resources table. This paper does not report original code. Any additional information required to reanalyze the data reported in this paper is available upon request.

## Supplemental information

Document S1. Figures S1–S4

Table S1. RNA-seq results of amdh-1(uth29) vs. wildtype animals, related to Figure 1

Table S2. Survival statistics of lifespan and stress resistance experiments, related to Figure 4
