# Supplementary material for: SKN-1 regulates stress resistance downstream of amino catabolism pathways
Source: iScience. 2022 Jun 9;25(7):104571. doi: 10.1016/j.isci.2022.104571 (PMC9240870; doi:10.1016/j.isci.2022.104571)
Supplement: Document S1. Figures S1–S4 [file mmc1.pdf]

iScience, Volume *25*

Supplemental information

SKN-1 regulates stress resistance

downstream of amino catabolism pathways

Phillip A. Frankino, Talha F. Siddiqi, Theodore Bolas, Raz Bar-Ziv, Holly K. Gildea, Hanlin Zhang, Ryo Higuchi-Sanabria, and Andrew Dillin

Figure S1. ***amdh-1*** **mutants do not upregulate** ***gcs-1*** **expression, Related to Figure 1**

(A) Schematic of *amdh-1/T12A2.1* genomic locus showing the location of allele *uth29* engineered using CRISPR/Cas9 (top, arrow). Scale bar, 100 bases. (B) Fluorescent images of SKN-1 reporter worms (*gcs-1p::GFP)* fed RNAi targeting *amdh-1* and *wdr-23*. Scale bar, 100 µm. (C) Quantification of (B)*,* Data shown are representative of n = 4 biological replicates with n > 60 animals per condition for each replicate. **** = P < 0.0001, n.s. = not significant using a one-way ANOVA non-parametric test (Kruskal-wallis).

Figure S2. ***gcn-2*** **and** ***let-363*** **are partially required for SKN-1 activation in** ***amdh-1*** **mutants, Related to Figure 2**

(A) Fluorescent images of SKN-1 reporter animals (*gst-4p::GFP)* fed RNAi targeting. Scale bar, 100 µm. (B) Quantification of (A), Data shown are representative of n = 2 biological replicates with n > 123 animals per condition for each replicate. (C) Table of causative mutations mapped using whole genome sequencing.

Figure S3. ***haly-1*** **is required for SKN-1 activation in** ***amdh-1*** **mutants, Related to Figure 3**

(A) Schematic of *haly-1* genomic DNA (gDNA) rescue array used for genetic rescue experiments to confirm causality (B) Fluorescent images of *haly-1; amdh-1* double mutants with or without *haly-1* gDNA rescue array. Scale bar, 100 µm.

Figure S4. Stress response pathways are not activated in *amdh-1* mutants, Related to Figure 4

(A) Fluorescent images of heat shock response (HSR) reporter animals (hsp-70p::GFP) with or without heat shock treatments (34C) in wildtype and amdh-1(uth29) mutant animals. Data shown are representative of n = 3 biological replicates. Scale bar, 100 µm. (B) Fluorescent images of a reporter of the unfolded protein response of the ER (UPR ER) animals (*hsp-4p::GFP)* fed RNAi. Scale bar, 100 µm. (C) Quantification of (B), Data shown are representative of n = 3 biological replicates with n > 50 animals per condition for each replicate. **** = P < 0.0001, n.s. = not significant using a one-way ANOVA non-parametric test (Kruskal-wallis) (D) Fluorescent images of UPR Mito reporter animals (*hsp-6p::GFP)* fed RNAi. Scale bar, 100 µm. (E) Quantification of (D), Data shown are representative of n = 3 biological replicates with n > 50 animals per condition for each replicate. **** = P < 0.0001, n.s. = not significant using a one-way ANOVA non-parametric test (Kruskal-wallis)

Figure S1

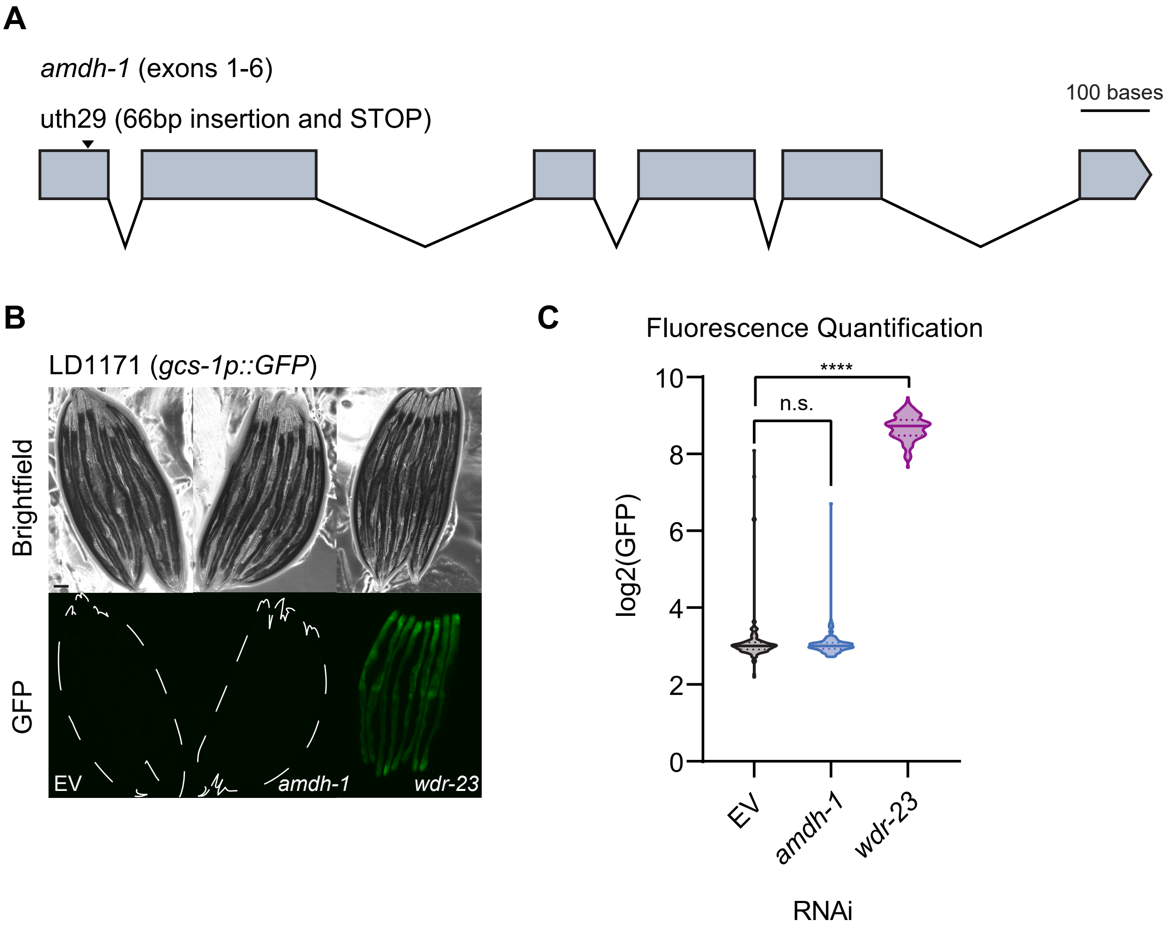

A
amdh-1 (exons 1-6)
uth29 (66bp insertion and STOP)
100 bases
B
LD1171 (gcs-1p::GFP)
Brightfield
GFP
EV
amdh-1
wdr-23
C
Fluorescence Quantification
****
n.s.
log2(GFP)
0
2
4
6
8
10
EV
amdh-1
wdr-23
RNAi

Figure S2

A

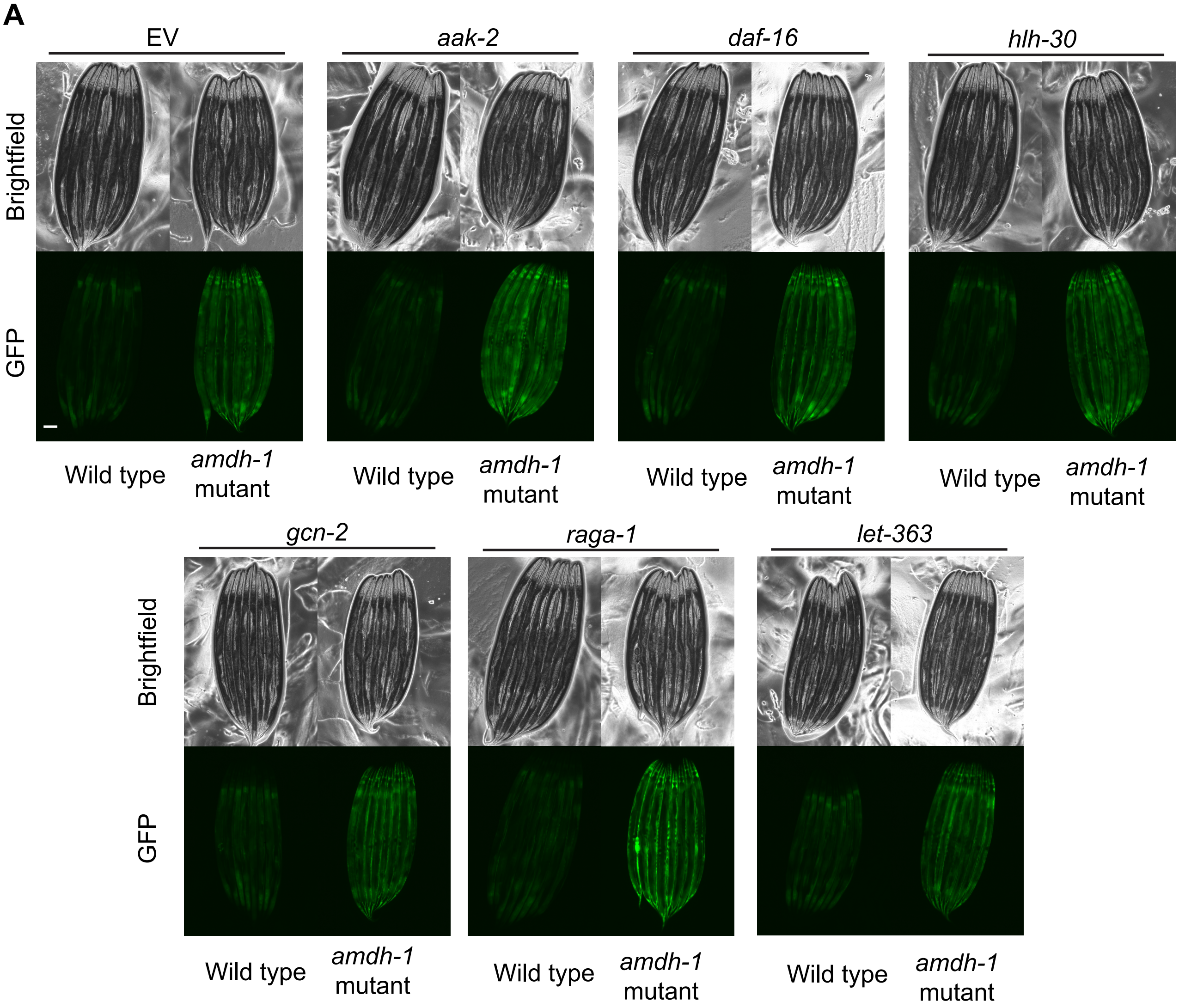

B

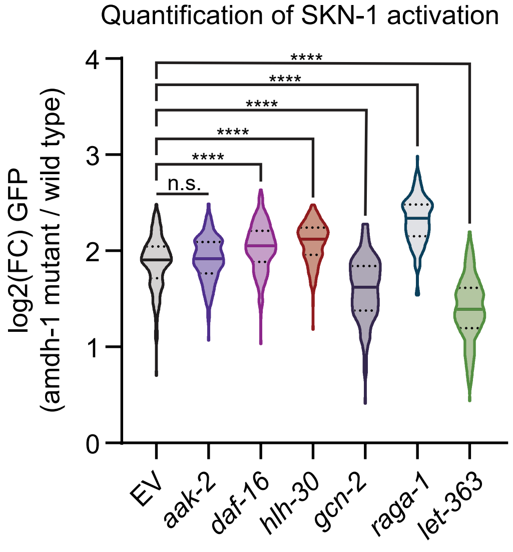

C

**Allele**	**Gene**	**Mutation**
uth89	*skn-1*	A514T
uth94	*elt-3*	Q128*
uth112	*suco-1*	A429M
uth92	*haly-1*	G134E
uth93	*haly-1*	G265E
uth95	*haly-1*	G551V

Figure S3

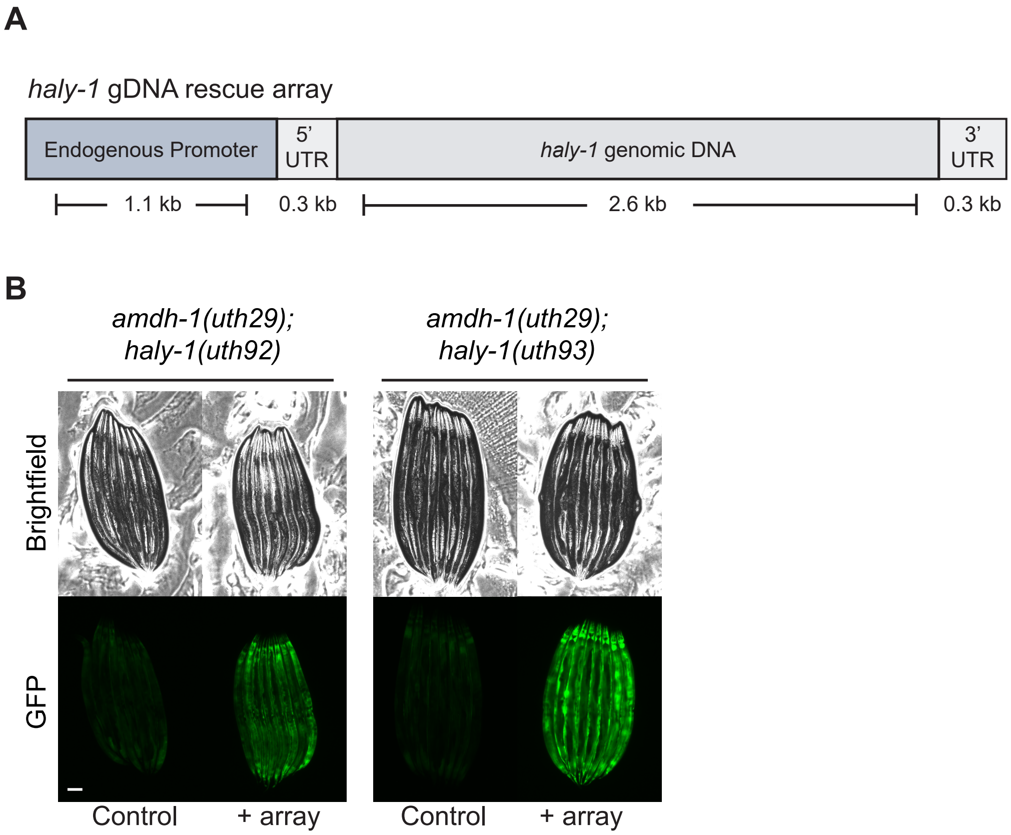
A
haly-1 gDNA rescue array
Endogenous Promoter
5' UTR
haly-1 genomic DNA
3' UTR
1.1 kb
0.3 kb
2.6 kb
0.3 kb
B
amdh-1(uth29); haly-1(uth92)
amdh-1(uth29); haly-1(uth93)
Brightfield
GFP
Control
+ array
Control
+ array

Figure S4

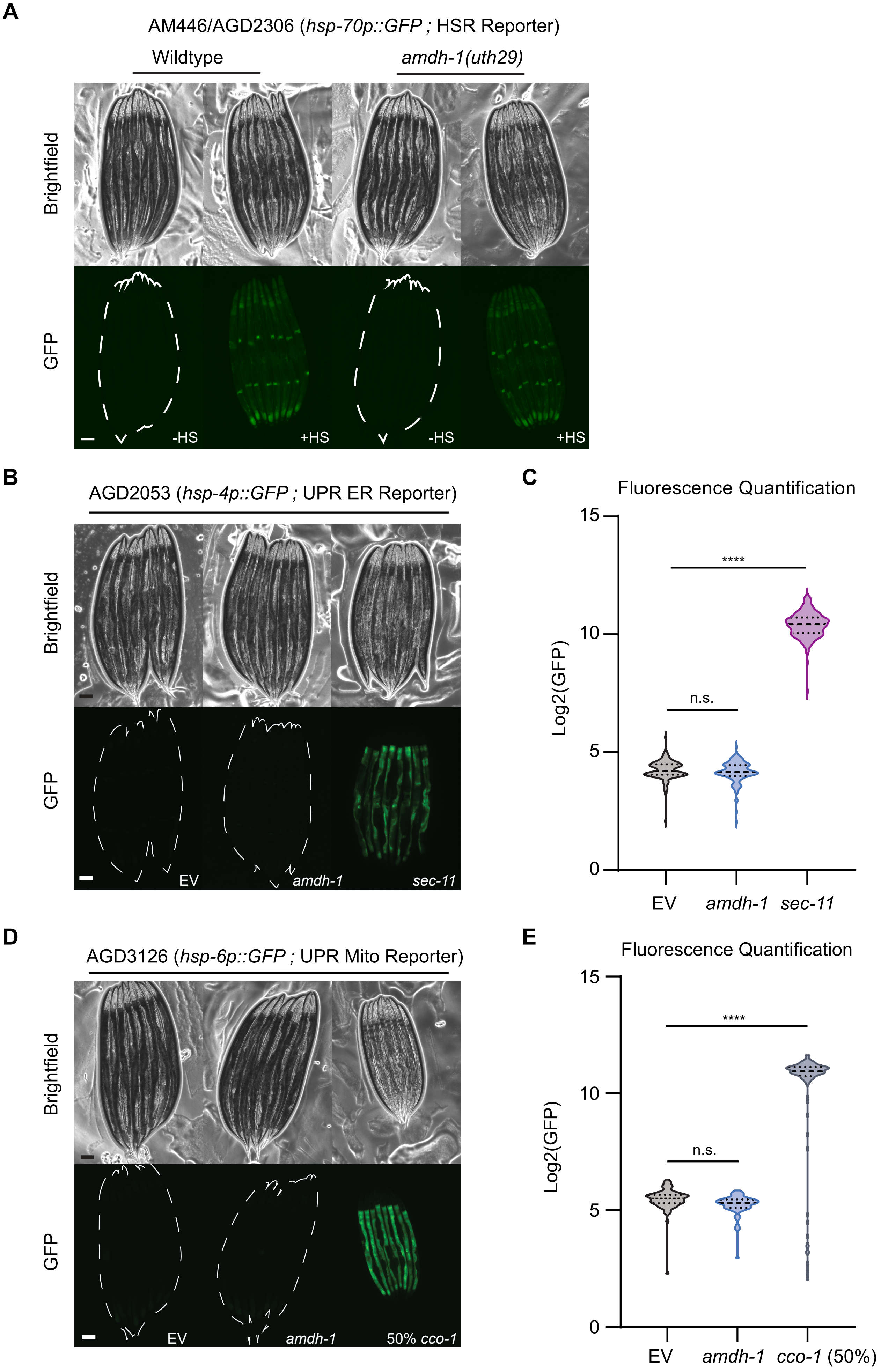
A
AM446/AGD2306 (hsp-70p::GFP ; HSR Reporter)
Wildtype
amdh-1(uth29)
Brightfield
GFP
-HS
+HS
-HS
+HS
B
AGD2053 (hsp-4p::GFP ; UPR ER Reporter)
Brightfield
GFP
EV
amdh-1
sec-11
C
Fluorescence Quantification
Log2(GFP)
15
10
5
0
****
n.s.
EV
amdh-1
sec-11
D
AGD3126 (hsp-6p::GFP ; UPR Mito Reporter)
Brightfield
GFP
EV
amdh-1
50% cco-1
E
Fluorescence Quantification
Log2(GFP)
15
10
5
0
****
n.s.
EV
amdh-1
cco-1 (50%)
